# Predictors of bullying reported by perpetrators in a sample of senior school students in Benin City, Nigeria

**DOI:** 10.4102/sajpsychiatry.v26i0.1359

**Published:** 2020-01-30

**Authors:** Oluyemi O. Akanni, Anthony A. Olashore, Samuel O. Osasona, Enobakhare Uwadiae

**Affiliations:** 1 Clinical Services, Federal Neuro-Psychiatric Hospital, Benin City, Nigeria; 2 Department of Psychiatry, Faculty of Medicine, University of Botswana, Gaborone, Botswana; 3 Department of Mental Health, University of Benin Teaching Hospital, Benin City, Nigeria

**Keywords:** bullying, cultism, predictor, prevalence, perpetrators, senior students, south-south, Nigeria

## Abstract

**Background:**

Bullying behaviour amongst adolescents is becoming a significant public health challenge. Whilst the traditional and electronic bullying as reported by victims has been widely reported, surveys amongst perpetrators, particularly in Africa, are still lacking.

**Aim:**

This study is aimed at determining the prevalence of bullying by perpetrators and analysing the relationship between bullying perpetration and psycho-socio-demographic characteristics amongst senior school students in Benin City, Nigeria.

**Setting:**

Senior secondary school in Benin City, Nigeria.

**Methods:**

A cross-sectional survey of 465 final-year secondary school students aged 16–19 years, who were selected by multistage random sampling, was conducted. The students were made to fill a self-designed questionnaire, in addition to an adapted version of the Wagnild and Young’s resilience scale.

**Results:**

The lifetime prevalence of bullying was 16.3%. Binary logistic regression revealed bullying to be significantly associated with students who are male (adjusted odds ratio [AOD] = 2.13, confidence interval [CI] = 1.16–3.93), have poor relations with their teachers (AOR = 2.98, CI = 1.68–5.29), have used alcohol (AOR = 3.51, CI = 1.74–7.09) and are involved in cult and gangsterism (AOR = 9.14, CI = 2.55–32.75).

**Conclusion:**

The rate of bullying perpetration by youth in Benin City, Nigeria, is significant and is comparable to global occurrence. The predictors of bullying in this study suggest that perpetrators are individuals who may benefit from rehabilitative measures.

## Introduction

Bullying behaviour amongst adolescents and students represents a significant public health challenge.^1^ Although the meaning of the term ‘bullying’ can vary according to culture,^2^ it is usually defined as a specific form of aggression which is intentional, repeated and involves a disparity of power between the victim and the perpetrator.^3^

Bullying is intended to harm or disturb a victim despite the victim’s apparent distress.^4^ It includes not only physical aggression (e.g. hitting, pushing and kicking) but also verbal aggression (e.g. name-calling, teasing^3^ and relational bullying such as social exclusion and spreading of rumours).^5^ The emergence of computers and cell phones heralded cyberbullying or electronic bullying, which has become popular amongst adolescents. It is a form of aggression that occurs through electronic mails, instant messaging or text messaging.^6^

Bullying has a popular image as a school-based behaviour; however, it can occur in other settings like lunchrooms, hallways, on the bus,^3^ gardens, parks,^7^ home, workplace and streets. Costa et al. reported that the highest frequency of bullying (55.1%) occurred in the school, and this might explain why most national and international studies have generally approached bullying using school survey.^8^

Reported prevalence rates of bullying across many studies and countries suggest that bullying is a distressingly common phenomenon amongst adolescent and student samples.^9^ Although prevalence rates have varied with the definitions of bullying, samples, ethnicity and measurement periods, prevalence data based on a large sample of students have reported reasonably and consistently high rates of bullying and victimisation despite methodological differences.^9^

Amongst a large sample of 202 056 school-aged children and adolescents in 40 countries worldwide, Craig et al. reported that the prevalence of being involved in bullying (as a bully, victim, or bully and victim) varied considerably between countries, with estimates ranging from 8.6% to 45.2% in boys and 4.8% – 35.8% in girls.^10^ In a sample of adolescents in the United States, Nansel et al. found that the prevalence of frequent involvement in school bullying in the past 2 months was 29.0%.^11^ In South America, researchers found that amongst a sample of 598 Brazilian adolescents aged 14–17 years, the prevalence of bullying was 26.4%,^8^ whilst in Venezuela 37.5% of male and 27.0% of female adolescents reported having been the victims of bullying at least once within the past 30 days.^12^

In Nigeria, a high prevalence of bullying has been similarly reported. It was found from a randomly selected sample of 300 junior secondary school students in Benin City, South-South Nigeria, that almost four in five participants (78%) reported being bullied, whilst 71% admitted that they had bullied someone at least once.^7^ Another study conducted in Ondo State, South-West Nigeria, with a sample consisting of 600 students selected through multistage sampling technique from six secondary schools, showed that 28% of the sample had experienced bullying, whilst 42% had bullied other students.^13^

The international literature reveals that bullying behaviours are universal and complex, and many personal and situational factors are associated with it.^9^ Many previous studies have reported that boys have a higher prevalence of bullying perpetration than girls.^7,10,13,14^ Researchers have also reported that boys are more involved in direct bullying, whilst girls are more involved in indirect bullying.^5,6,15^ Direct bullying involves obvious, unadulterated forms of aggression towards the victim. In this type of bullying, the bully usually does not conceal his or her identity and actions from the victim, whilst the reverse is often the case in the indirect type. Typically, indirect type of bullying occurs when an individual or a group of persons undermines or tries to ruin the victim’s reputation by spreading rumours or gossip about him or her. Although bullies are most often men, both men and women tend to be victims.^16^

Bullying is most common in late childhood, peaking at about 12 years of age with a transition to high school and then declining after that.^9^ Similarly, Pellegrini and Long in their study confirmed that the rates of bullying increased with a transition between schools because of a desire to establish social dominance amongst a new cohort of peers.^17^ Nansel et al.^11^ reported that grades 5–8 had been consistently found to be the grades at which bullying is most likely to take place. They attributed this peak to the need for youths, at this age, to find ways of dealing with the changes in the format of their education.^11^

Previous studies have also reported that there is a difference between the rates of bullying and victimisation by different ethnic groups within the same country.^11^ Although it is not yet clear, factors that are related to the ethnic differences and socio-economic status have been suggested as possible reasons.^9^ Many other factors found to be associated with bullying include family dynamics, rearing practices, interpersonal relationship, social context and use of psychoactive substances.^18,19^

Involvement in bullying, either as a perpetrator or as a victim, is associated with numerous physical, mental and social consequences.^20^ Perhaps because it is a distressing experience and often continues over the years, it has been found to predict both concurrent and future psychiatric symptoms and disorders, such as attention deficit hyperactivity disorders and anxiety at young ages, personality disorders and anxiety in young adults.^21^ Other studies and systematic reviews have shown that victims of bullying have lower self-esteem, suffered more from depression and stress and were more likely to think about or attempt to self-harm and suicide than non-victims.^22,23^ Also, a relationship exists between a student’s bullying behaviour and school issues such as impairment in the academic progress of victims.^20^

Despite the relatively high prevalence of bullying across studies and countries and the enormous consequences that are associated with it, it is under-researched in Nigeria. Most studies on bullying have been conducted in the developed countries; the few available studies in Nigeria have focussed on the victims of bullying, and little profiling has been carried out on perpetrators. Thus, it is crucial to investigate bullying from the end of the culprit in the Nigerian setting. This finding will add to the body of literature by filling in gaps in the understanding of the risk factors of bullying within the Nigerian context. Moreover, the finding will contribute to preventive measures of bullying because identification and handling of bullies will lead to a safe environment for the vulnerable ones. Therefore, this study aimed to determine the prevalence of bullying as reported by perpetrators and examine the association of bullying amongst senior school students with demographics, family, school-related, risky behaviours and psychological characteristics.

## Methods

### Study location

The study was a cross-sectional study carried out in Benin City, South-South Nigeria. The city is situated within three local government areas (LGAs): Egor, Ikpoba-Okha and Oredo. The selected LGA (Oredo) has 138 secondary schools comprising 13 government-owned (public) and 125 government-approved private schools with student populations of 23 672 and 38 688, respectively. Thus, the public-to-private school students ratio is 1:1.6.

### Study population

The study sample consisted of final-year adolescent students, aged 16–19 years, in the senior secondary schools (SSS). The choice of students was informed based on various considerations that include the ability to comprehend contents of the test materials, the cut-off age for the chosen instrument and to make for easy comparison of findings with existing studies in the literature.

### Sampling technique

A multistage random sampling technique was adopted in selecting the final-year students of the SSS. The first stage involved a random selection of Oredo LGA. In the second stage, 18 gender-mixed schools from the same LGA were selected by stratifying the schools into public and private schools to ensure appropriate school representation. The selection was restricted to only mixed-gender schools because all the private schools in Oredo LGA are mixed-gender schools, and unregistered schools were excluded from the process. At this stage, four public and 14 private schools were chosen. In the last stage (third), students were selected from each of the participating schools using the proportional representation method. A simple random sampling technique was applied using the class register to select the students from each arm of the final-year classes. A total of 187 students from the public and 305 from the private schools were recruited to maintain the ratio of 1:1.6, thus making a total sample size of 492 students. This figure exceeded the minimum estimated sample size of 317 students calculated when the Fisher’s formula was applied^24^ using 71% from a previous study as a lifetime prevalence rate of bullies^7^ and probability of type 1 error margin of 5%.

### Selection criteria

Students in the SSS three classes outside the age range of 16–19 years and those who or whose parents or guardians refused to give consent were excluded from the study.

### Questionnaire administration

The questionnaires were administered to students in their classrooms during break time in batches of 15–30 students with the help of two trained research assistants. The students required minimal assistance, which was provided, for filling in the self-report questionnaire. During the process, confidentiality in handling information was strictly observed. The duration of the study lasted for six and a half weeks.

### Measures

#### Demographics

These included gender, age and religion. Age was categorised into 16–17 and 18–19 years groups, whilst religion was dichotomised into Christianity and other religion because Christianity was the dominant religion in the area.

#### Family-related factors

These factors covered family structure, parental religiosity and perceived relationship with parents. Parental religiosity as perceived by the respondents was rated as either high if parents or guardians pray regularly or low if they occasionally or hardly pray.

#### School and academic-related factors

Enquiries were made regarding the school type, perceived relationship of participants with teachers and academic performance. Academic performance was investigated as a ‘yes or no’ question by asking whether respondents had repeated a class in the past.

#### Psychological factors

Feeling of stress and psychological resilience were examined. The resilience scale developed by Wagnild and Young was used to measure the degree of each participant’s resilience;^25^ however, the scale has been adapted for use in a previous study amongst Nigerian school students.^26^ The adapted version, which has a Cronbach’s alpha of 0.65 and recorded a significant correlation with the original scale (*r* = 0.74, *p* = 0.00), was used in this study. Responses were made on a seven-point scale, with higher scores indicating greater resilience; however, the participants were classified into either low or high resilience based on the mean score of 34.9.

Inquiries about risky behaviours such as involvement in cult and gangsterism, alcohol, tobacco and cannabis use were made on whether the behaviour had ever been carried out in the past.

Bullying was enquired through a ‘yes or no’ question. The operational definition of bullying covers any form of bullying ever inflicted on someone in school, such as kicking, punching, slapping, gossiping, name-calling, threatening, intimidating and teasing.

### Statistical analyses

Data were analysed using the Statistical Package for Social Sciences (SPSS) 22. A frequency distribution was performed to determine the prevalence of bullying. Continuous variable such as age was categorised into two groups: 16–17 and 18–19 years. Chi-squared test was used to determine the variables associated with bullying. Furthermore, variables significantly associated with bullying on bivariate analysis were subjected to binary logistic regression to determine the predictors of the same. A *p*-value of less than 0.05 was set as statistical significance.

### Ethical considerations

Ethical approval was obtained from the Ethics Committee of the University of Benin Teaching Hospital. Permission was also obtained from the Ministry of Education and the authorities of the schools involved. The students who were recruited to fill the questionnaires for the study either obtained written consent from their parents or guardians if they were less than 18 years or gave their consent if they were 18 years and above. The consent form was given to those less than 18 years to take home to their parents or guardians and to return the following day.

## Results

A total of 492 questionnaires were returned following administration; 27 questionnaires were discarded because they were either incompletely filled or contained inconsistent responses, or both. The proper response rate of 94.5% and 465 questionnaires were analysed. The mean age of the respondents was 16.9 years (standard deviation [SD] = 0.9 year).

There were 210 (45.2%) female and 255 (54.8%) male students; of the students, 183 (39.4%) were in public schools and 282 (60.6%) were in private schools.

About 16.3% of students reported bullying someone in school in their lifetime. Being a male, a product of a polygamous family, low parental religiosity, history of having ever used alcohol, tobacco and cannabis, having repeated a class, lifetime history of involvement in cultism and gangsterism and not having a satisfying relationship with teachers were all significantly associated with bullying someone (see Tables 1 and 2). Following a binary logistic regression, the independent predictors of bullying perpetration were being a male, having ever used alcohol, involvement in cult and gangsterism, and a lack of satisfactory relationship with teachers. Male students were two times more likely (adjusted odds ratio [AOD] = 2.13, confidence interval [CI] = 1.16–3.93) to bully compared to their female counterparts. Students who had used alcohol reported three times more likelihood of bullying than those who had never used, whilst those who were involved in cult and gangsterism had about nine times increased rate of bullying than their counterparts who had not done so. Adolescents who reported ‘no’ satisfaction about the relationship with their teachers also predicted bullying (AOR = 2.73, CI = 1.50–4.95). Students who reported past use of tobacco and cannabis fell short of statistically significant relationship with bullying (*p* = 0.05 and 0.07, respectively) (see Table 3).

**TABLE 1:** Association of bullying with demographics, family and school-related factors.

Variables	Bullying						Significant test		
	No (*n* = 389)		Yes (*n* = 76)		Total (*n* = 465)		χ^2^	df	*p*
	*n*	%	*n*	%	*n*	%			
**Gender**							11.27	1	0.00
Male	200	51.4	55	72.4	255	54.8	-	-	-
Female	189	48.6	21	27.6	210	45.2	-	-	-
**Age (years)**							1.45	1	0.22
16–17	306	78.7	55	72.4	361	77.6	-	-	-
18–19	83	21.3	21	27.6	104	22.4	-	-	-
**Religion**							1.13	1	0.29
Christian	370	95.1	70	92.1	440	94.6	-	-	-
Others	19	4.9	6	7.9	25	5.4	-	-	-
**Family**							4.13	1	0.04
Monogamous	312	80.2	53	69.7	365	78.5	-	-	-
Polygamous	77	19.8	23	30.3	100	21.5	-	-	-
**Parental religiosity**							6.98	1	0.00
High	314	80.7	51	67.1	365	78.5	-	-	-
Low	75	19.3	25	32.9	100	21.5	-	-	-
**Satisfaction with parents**							3.25	1	0.07
No	51	13.1	16	21.1	67	14.4	-	-	-
Yes	338	86.9	60	78.9	398	85.6	-	-	-
**School**									
Public	160	41.1	23	30.3	183	39.4	3.15	1	0.08
Private	229	58.9	53	69.7	282	60.6	-	-	-
**Repeat a class**							4.67	1	0.03
No	339	87.1	59	77.6	398	85.6	-	-	-
Yes	50	12.9	17	22.4	67	14.4	-	-	-
**Satisfaction with teachers**							22.20	1	0.00
No	69	17.7	32	42.1	101	21.7	-	-	-
Yes	320	82.3	44	57.9	364	78.30	-	-	-

**TABLE 2:** Association of bullying with psychological factors and risky behaviours.

Variables	Bullying						Significant test		
	No (*n* = 389)		Yes (*n* = 76)		Total (*n* = 465)		χ^2^	df	*p*
	*n*	%	*n*	%	*n*	%			
**Feeling of stress**							0.72	1	0.40
Not at all	43	11.1	11	14.5	54	11.6	-	-	-
Sometimes-always	346	88.9	65	85.5	411	88.4	-	-	-
**Psychological resilience**							0.01	1	0.92
Low	151	38.8	30	39.5	181	38.9	-	-	-
High	238	61.2	46	60.5	284	61.1	-	-	-
**Cannabis use**							15.88	1	0.01*
No	384	98.7	69	90.8	453	97.4	-	-	-
Yes	5	1.3	7	9.2	12	2.6	-	-	-
**Alcohol use**							29.51	1	0.00*
No	193	49.6	12	15.8	205	44.1	-	-	-
Yes	196	50.4	64	84.2	260	55.9	-	-	-
**Tobacco use**							31.54	1	0.00*
No	366	94.1	56	73.7	422	90.8	-	-	-
Yes	23	5.9	20	26.3	43	9.2	-	-	-
**Gangsterism and cultism**							41.70	1	0.00*
No	385	99.0	64	84.2	449	96.6	-	-	-
Yes	4	1.0	12	15.8	16	3.4	-	-	-

* , Significant factors.

**TABLE 3:** Logistic regression of predictors of bullying.

Variables	B	SE	Wald	df	Sig.	AOR	95% CI
Gender	0.75	0.31	5.90	1	0.02*	2.13	1.16–3.93
Parental religiosity	0.20	0.32	0.40	1	0.53	1.23	0.65–2.30
Repeat a class	0.45	0.36	1.53	1	0.22	1.56	0.77–3.17
Family	0.36	0.33	1.16	1	0.28	1.50	0.75–2.76
Relationship with teachers	1.00	0.30	10.81	1	0.00*	2.73	1.50–4.95
Cultism and gangsterism	2.21	0.65	11.56	1	0.00*	9.14	2.55–32.75
Tobacco use	0.77	0.40	3.79	1	0.05	2.16	1.00–4.71
Alcohol use	1.25	0.36	12.28	1	0.00*	3.51	1.74–7.09
Cannabis use	0.59	0.76	0.61	1	0.43	1.81	0.41–7.93

AOR, adjusted odds ratio; CI, confidence interval; SE, standard error; Sig., significance.

* , Significant factors.

## Discussion

Traditional and electronic bullying, as reported by victims, has been widely reported,^27,28,29^ whereas surveys amongst perpetrators, particularly in Africa, are lacking. This study explored the characteristics of perpetrators of bullying amongst senior school students in a city in South-South, Nigeria. The lifetime prevalence of bullying as reported by the perpetrators in this community representative sample of Nigerians aged 16–19 years was 16.3%.

This rate is comparable with 13% reported amongst school children in South Africa,^28^ 14% amongst autistic children in the United States^30^ and 15% from a systematic review.^31^ These figures thus suggest that it is a global problem and not restricted to any one part of the world. Whilst most of the reports from the developed countries are on the cyber and electronic form of bullying,^29,31,32^ our study included methods of bullying applied in school, including physical and verbal bullying, which is believed to be more common. Perhaps this may be because information technology is not as popular amongst our youths compared to the developed world. As in other parts of the world, physical bullying in Nigeria encompasses an imbalance of control that is exhibited in acts of physical or interpersonal aggression.^11^ This has a severe consequence for the victim’s life, and it is associated with many psychiatric conditions and poor academic performance of the victims.^4,33,34^ In this respect, measures to prevent bullying in schools are necessary.

Women are likely to be at the receiving end of violence or bullying, whereas men are more likely to be the perpetrators.^4,34^ Although our study did not include victims, the finding of a significant association between being a male and bullying perpetrator was replicated in the current study after logistic regression analysis.

Studies have substantiated the age-long lay assumptions that violence and aggressive behaviour are associated with being male.^35^ The link, although debatable, has been explained by the Y-chromosomes.^36^ Differential exposure to androgens in the prenatal life was found to be related to a gender difference in mind dynamics, altruistic cooperativeness, emotional sensitivity and risk-taking behaviours.^37,38^ This explains to an extent the difference in temperament observed in the two genders in the early periods of life.

In addition to the biological factors, the effects of life experiences and environmental influences on the mind have been used to explain the gender difference in bullying and negative attitudes towards peers.^39^ Men and women are educated customarily to carry out different social roles; for example, parental nurturing of daughters versus sons differs across cultures.^35^ By tradition, mothers tend to share their emotional feelings and experience with their daughters, and they are more often taught to look after or care for others.^35^ This upbringing tends to increase the awareness of their inner world and those of others.^35^ Consequently, girls are more likely to engage in self-absorption or ruminative self-focus and self-directed aggression compared to boys who by nurture might prefer a more outward-directed method of aggression.^35^ In African settings, where more priority is placed on a male child, both parents are likely to encourage the aggressive and domineering roles of boys. For instance, parents buy toy guns for boys, which signifies or encourages violence, and dolls for girls, which signifies a passive and caring role. They often encourage boys to stand up to their peers compared to girls who are trained to be tolerant and caring. These practices may explain why males are predominant perpetrators of bullying and females are usually at the receiving end.

In this study, adolescents who are bullies were found to have increased risk of alcohol use compared with those with no bullying experience. It has also been reported that adolescents who have experienced bullying, whether as victims or as perpetrators, have an increased risk of drinking compared with those with no bullying experience.^40^ Abnormal use of alcohol possesses the ability to lower inhibitions, impair judgement and increase the risk of violence and aggressive behaviour, especially in those who are timid but possess an aggressive tendency.^41^ Besides, bullying perpetration and substance use may share a common origin; for example, some personality dimensions such as impulsivity, which were not explored in this study, may predispose to both bullying and substance use.^42^ It is thus not surprising in this present study to find a strong relationship between alcohol use and bullying after a regression model.

Lifetime use of cannabis was not found to predict bullying in this study independently, but it has a significant association with it, as shown in previous reports.^29,31^ Although the relationship between substance use and bullying is not fully understood, a drug such as cannabis has an effect of reducing inhibition and causing a false sense of confidence or being in control. People who use cannabis are likely to be paranoid, misread actions which are harmless as threats and therefore might become aggressive towards people around them.^43,44^ It is thus not unusual to find adolescents using drugs like cannabis, bullying or being aggressive towards peers. This critical factor in bullying suggests the need to also offer treatment to the perpetrators in addition to the victims.

However, when other variables such as age, gender and polygamous family that are known to be associated with cannabis use in this environment^45,46^ were controlled for in a logistic regression analysis, its contribution to bullying in our sample paled out.

The present study highlights the role of gangsterism in bullying perpetration. Being a member of a gang increased the rate of bullying by nine times when compared to students who were not members of such a group.

Gangsterism has been previously related to bullying, perpetration and victimisation.^32,47^ In the Nigerian context, some authors have described gangsters as ‘secret cult or sect,’ and a group of delinquent youths who are armed with guns or group of students acting as terrorists or bullies within the school system.^48^ Generally, cultism is understood as activities similar to gangsterism, as against its meaning of a religious sect with extreme views or small group of devoted supporters. These two terms have been used interchangeably in many literature in Nigeria to refer those who clash with accepted norms and values of everyday life.^48,49^ The activities of these groups of persons are usually carried out at unusual times, and they include, but not limited to, destruction of private and public property, disruption of academic activities in schools, rape, substance abuse and bullying.^48,49^ In the context of psychiatric diagnosis, cultism in Nigeria can be linked to socialised conduct disorder, as the majority of these individuals manifest most of the criteria listed in the International Classification of Diseases (ICD-10).^50^ A proportion of these individuals may have been victims of bullying and only joined the gang for protection against bullying and intimidation, whilst some may become members because of a steady supply of psychoactive drugs.^48,49^ They are usually bound by oath to carry out the mission of the gang even if it is against their wishes,^49^ thus suggesting that perpetrators of bullying in this regard need help as much as the victims.

Adolescent bullies are likely to perceive the relationship with their teachers negatively, as suggested by the present study. This negative perception may be related to being in the ‘bad book’ of their instructors. Lyznicki et al. have suggested that bullies have trouble following rules, and do not often have a good relationship with their teachers and parents.^4^ They are at increased risk of poor school performance, failure and school dropout. Although poor academic performance may be a consequence of antisocial behaviour, it may also explain why some of these adolescents engage in antisocial behaviour as a way of coping with low self-esteem and lack of confidence associated with poor school performance. These facts again reiterate the need to see these individuals as children in need of help and protection, rather than those who deserve punishment and expulsion. Bullies need psychological interventions to develop adaptive ways of coping with problems, to deal with drugs and to learn healthy ways to interact with peers. Professional help can make them see the harm in bullying others and become sensitive to people and understand the feelings of others.^34^ Focussing on them has an added advantage of indirectly affecting victims because it will promote a safe environment for the vulnerable students. Thus, a holistic approach of paying attention to the needs of perpetrators, in addition to the rehabilitative effort provided to victims, maybe a better method of targeting the menace of bullying and ultimately eradicating it from schools.

## Limitations and strengths

This study has some limitations that must be discussed. Firstly, the sample size was small and the study was conducted in only one major city in Nigeria. This limits the generalisability of the findings to other parts of the country and obliterates the possibility of identifying cultural influences on bullying. Secondly, the study did not include electronic bullying and therefore should be interpreted with caution. The study was focussed on bullying carried out in the school premises; hence, it did not cover cyberbullying. Further study should investigate this because it is an essential form of bullying. Also, there is a section of private secondary schools in Benin City that is blind to the authorities. These schools are cheap and unregistered. They thrive because of the poor financial fortunes of the average Nigerian that serve as an attraction to patronise substandard schools. These illegal schools were not captured in this work. A definite statement on the state of bullying amongst Nigerian students may need to take this into account in the future. Lastly, predictability is difficult with a cross-sectional study because cause and effect cannot be precisely ascertained. However, the study possesses some strengths worth mentioning. It looked at bullying from the angle of the perpetrators, especially in this part of the world where less attention is still being paid to children in need of care, who are often labelled ‘beyond parental control’.^51^ The study investigated numerous factors suspected to be associated with bullying and looked across varying socio-economic strata by involving students from both private and public secondary schools.

## Conclusion

The rate of bullying perpetration by youths in Benin City, Nigeria, is significant and the pattern follows the prevalence that has been reported elsewhere, with men dominating the world of bullying perpetration. The association between bullying and identified variables, such as cultism which may indicate an antisocial problem and poor relationship with teacher, suggests the need to provide care and protection rather than punishment and expulsion to perpetrators of bullying. The burden it poses on individual health of both the victim and the perpetrator may just yet not have been transmitted to a healthcare burden because of lack of awareness and under-reportage.
